# Polyploid Adipose Stem Cells Shift the Balance of IGF1/IGFBP2 to Promote the Growth of Breast Cancer

**DOI:** 10.3389/fonc.2020.00157

**Published:** 2020-02-18

**Authors:** Roberta Fajka-Boja, Gábor J. Szebeni, Éva Hunyadi-Gulyás, László G. Puskás, Róbert L. Katona

**Affiliations:** ^1^Artificial Chromosome and Stem Cell Research Laboratory, Biological Research Centre, Institute of Genetics, Szeged, Hungary; ^2^Laboratory of Functional Genomics, Biological Research Centre, Institute of Genetics, Szeged, Hungary; ^3^Department of Physiology, Anatomy and Neuroscience, Faculty of Science and Informatics, University of Szeged, Szeged, Hungary; ^4^Laboratory of Proteomics Research, Biological Research Centre, Institute of Biochemistry, Szeged, Hungary; ^5^Avidin Ltd., Szeged, Hungary

**Keywords:** adipose stem cells, breast cancer, tumor stroma, insulin-like growth factor 1, insulin-like growth factor binding protein 2

## Abstract

**Background:** The close proximity of adipose tissue and mammary epithelium predispose involvement of adipose cells in breast cancer development. Adipose-tissue stem cells (ASCs) contribute to tumor stroma and promote growth of cancer cells. In our previous study, we have shown that murine ASCs, which undergo polyploidization during their prolonged *in vitro* culturing, enhanced the proliferation of 4T1 murine breast cancer cells in IGF1 dependent manner.

**Aims:** In the present study, our aim was to clarify the regulation of ASC-derived IGF1.

**Methods:** 4T1 murine breast carcinoma cells were co-transplanted with visceral fat-derived ASCs (vASC) or with the polyploid ASC.B6 cell line into female BALB/c mice and tumor growth and lung metastasis were monitored. The conditioned media of vASCs and ASC.B6 cells were subjected to LC-MS/MS analysis and the production of IGFBP2 was verified by Western blotting. The regulatory effect was examined by adding recombinant IGFBP2 to the co-culture of ASC.B6 and 4T1. Akt/protein kinase B (PKB) activation was detected by Western blotting.

**Results:** Polyploid ASCs promoted the tumor growth and metastasis more potently than vASCs with normal karyotype. vASCs produced the IGF1 regulator IGFBP2, which inhibited proliferation of 4T1 cells. Downregulation of IGFBP2 by polyploidization of ASCs and enhanced secretion of IGF1 allowed survival signaling in 4T1 cells, leading to Akt phosphorylation.

**Conclusions:** Our results implicate that ASCs in the tumor microenvironment actively regulate the growth of breast cancer cells through the IGF/IGFBP system.

## Introduction

Nowadays, stem cell-based therapies are feasible tools for treatment of various diseases. Adipose-tissue derived stem cells (ASCs) are especially popular, as these cells can be easily harvested in large quantities. ASCs have a great potential to proliferate, to differentiate into various cell lineages and to modulate immune responses, therefore they seem to be ideal for the treatment of orthopedic or inflammatory diseases (1). ASCs have multiple roles in regeneration: they provide source for cell-renewal, and secrete paracrine factors for cell growth, revascularization, immunosuppression, and wound-healing. *In vivo* studies suggest that ASCs may be used for the treatment or adjunctive therapy for multiple sclerosis, ischemic stroke, glioblastoma, spinal fusion, chronic liver failure, acute kidney injuries, myocardial ischemia, chronic obstructive pulmonary disease, osteoarthritis, and inflammatory bowel disease (1, 2). However, fewer studies reach clinical phase II or beyond, and the first marketing authorization of an allogeneic stem cell therapy was approved in 2018 for the treatment of complex perianal fistulas in Crohn's disease (3). Although ASCs are considered to be panacea, the U.S. Food and Drug Administration (FDA) warns that only those therapies are acceptable, which are proved to be safe and efficient in randomized, controlled trials (4). Many clinics use the so called stromal vascular fraction (SVF), isolated in a single step from the autologous adipose tissue (5). This method avoids cell expansion *in vitro*; however, it also escapes careful characterization of the utilized stem cell product. SVF consists of heterogeneous cell population, depending on donor age, gender, and weight, and the anatomic harvest location, which causes high variation in clinical outcome. The better approach would be establishment of an ASC bank, as ASCs are not immunogenic, and cells from allogeneic source can be used for therapies (6). The cells from an ASC bank could be expanded *ex vivo* and fully characterized prior to clinical use. An emerging problem with the *ex vivo* expanded stem cells is that they show chromosomal instabilities (7–9), which may be associated with cancer. Moreover, ASCs have been shown to integrate into tumor microenvironment, where they may promote the tumor progression by direct cell-cell contact or paracrine factors (10–12).

In our previous study, we have shown that murine ASCs became hypotetraploid under prolonged *in vitro* culturing, which was accompanied with phenotypical, gene expressional and functional changes. Polyploid ASC.B6 cells upregulated the expression of several stemness factors, such as Krueppel-like factor 4 (KLF4), and secreted growth factors, such as Insulin-like growth factor 1 (IGF1). We detected that ASC.B6 enhanced the *in vitro* proliferation of 4T1 murine breast cancer cells in an IGF1-dependent manner (13). IGF1 is crucial during mammary gland development; however, it also plays important role in breast cancer (14). It is produced in the liver and transported via blood into various tissues in the body, bound to members of the Insulin like growth factor-binding protein family (IGFBPs). The six members of this family bind IGFs with high affinity, and as they are expressed in most tissues, they play important role in the regulation of IGF activity both on endocrine and autocrine/paracrine levels (15). The importance of the IGF/IGFBP system in cancer progression has been emphasized recently: IGFs are autocrine factors for many cancers, while IGFBPs hinder tumor growth by inhibiting IGF functions, such as cell proliferation, survival, and migration/invasion. The balance of these proteins is often perturbed in malignant diseases, including glioma, prostate, breast, and ovarian cancer, although the tumor suppressor function of IGFBPs in individual cases is often debated (16). Given that we have found upregulated IGF1 production by polyploid ASCs, which promoted breast cancer cell proliferation *in vitro*, we aimed to confirm the tumor-promoting function of these stem cells and to clarify the underlying mechanism. In this study, we co-transplanted female mice with 4T1 breast cancer cells and ASCs with normal or polyploid karyotype and monitored tumor initiation, progression and metastasis. We detected that ASC.B6 cells downregulated their IGFBP2 expression in parallel with IGF1 upregulation, therefore we tested if the ASC.B6-induced 4T1 cell proliferation is influenced by adding recombinant IGFBP2. Finally we examined the PI3K/Akt pro-survival signaling pathway in 4T1 cells in the presence of ASC.B6-derived factors and IGFBP2.

## Materials and Methods

### Cells

Adipose stem cells (vASC) were isolated from visceral fat tissues of 4 months old mice, males and females, C57BL/6J strain (JAX). ASC.B6 cell line was established as previously described (13). ASC.B6 and vASCs were cultured in DMEM/F12 supplemented with L-glutamin (Gibco), Penicillin/Streptomycin (Sigma-Aldrich), 10% fetal calf serum (FCS, Gibco) and 5% horse serum (Gibco), at 37°C and 5% CO_2_. Conditioned media were prepared as follows: confluent cell cultures of ASC.B6 or vASCs were washed in PBS and kept in serum-free DMEM/F12 for 48 h. Then the supernatants were collected and centrifuged for 10 min at 300 × g to remove cell debris. 4T1 mouse breast carcinoma cells (ATCC CRL-2539) were maintained in DMEM/F12 with 5% FCS.

### Murine Breast Cancer Model

Female Charles River-derivative BALB/c mice (Balb-c/kby) (8–10 weeks old) were purchased from Kobay Ltd. (Turkey) and were injected orthotopically with 4T1 breast carcinoma cells (10^3^ cells/animal) with or without vASCs or ASC.B6 cells (10^5^ cells/animal). The animals had free access to food and water. Six mice were included into each experimental group. The experiments were repeated independently two times under same conditions and the pooled results have been presented in the paper. The incidence of palpable tumors was determined by regular monitoring of animals, and the tumor size was measured with a precision caliper and calculated according to the formula: d^2^ × D × 0.5, where d and D are the minor and major diameters, respectively. The experiment was terminated with euthanizing the animals on day 28, and then the primary tumors and lungs were excised and fixed in 4% formaldehyde fixation (Molar Chemicals, Budapest, Hungary). Weights of the primary tumors and lungs were measured after fixation. Mice showing signs of suffering (lost 15% of body weight and/or righting reflex is not working and/or enable to eat, drink) due to (ethical) legislation were sacrificed before terminating the experiment. Survival of experimental animals was estimated using Kaplan-Meier plot analysis.

### Protein Identification by LC-MS/MS

Conditioned media were harvested from confluent cell cultures and the proteins were precipitated with 10% trichloroacetic acid (TCA, Sigma), washed with acetone, and then boiled in sample loading buffer. The proteins were separated on a 15% SDS-PAGE and stained with Coomassie Brilliant Blue R-250 (Bio-Rad Laboratories). The dominant band between 28 and 36 kDa was cut and subjected to protein identification. The proteins were in-gel digested according to the protocol of the UCSF MS-Facility (http://ms-facility.ucsf.edu/protocols.html). Briefly: after reduction with 1,4-dithiothreitol [(DTT), Sigma] and alkylation of the free sulfhydryls with iodoacetamide [(IAM) Sigma] the proteins were digested with trypsin (Sequencing grade modified trypsin, Promega). Tryptic peptides were extracted and the digests were subjected to LC-MS/MS analysis using an LTQ-Orbitrap Elite (Thermo Fisher Scientific, Germany) mass spectrometer online coupled with a nanoAcquity-UPLC (Waters, USA) system. Peak picking was done using PAVA script and the peaklists were subjected to database search on our in-cloud ProteinProspector (*v.:5.20.0*) search engine using the mouse sequences of the UniProtKB.2017.9.19.random.concat (84204/89951742 entries searched) database.

### IGFBP2 Detection With Western Blotting

For detection of IGFBP2 from the cell lysates, the cells were detached and washed in PBS then counted. Cell concentration was adjusted with sample loading buffer (62.5 mM Tris HCl, pH 6.8, 2% SDS, 10% glycerol, 5% 2-mercaptoethanol, and 0.002% bromphenol blue) to 10^6^ cells/ml, boiled for 5 min and then vortexed for 1 min. Boiling and vortexing was repeated three times. Samples were centrifuged for 1 min at 13,000 × *g*. For detection of secreted IGFBP2 conditioned media were harvested from confluent cell cultures and the proteins were precipitated with 10% trichloroacetic acid (TCA), washed with acetone, and then boiled in sample loading buffer. Cell lysates of 10^5^ cells and precipitated proteins from 1 ml of conditioned media were run on a 10% SDS-PAGE. The proteins were transferred to polyvinylidene difluoride membranes (Immobilon-P PVDF, Millipore). The membranes were blocked with 3% gelatin from cold-water fish skin (Sigma) in PBS for 1 h at room temperature, and then incubated with anti-IGFBP2 antibody (Santa Cruz Biotechnology, sc-515134) overnight at 4°C. After washing and incubating with swine anti-rabbit Ig-HRP (DAKO) for 1 h at room temperature, the immunoreactive proteins were visualized using WesternBright ECL HRP substrate (Advansta), and the chemiluminescence signal was detected with Odyssey Imaging System (LI-COR Biotechnology). Rabbit anti-β actin antibody (Abcam, ab8227) and anti-rabbit Ig-HRP was used to test the equal amount of loaded proteins.

### Phospho-Akt Stimulation and Western Blotting

4T1 cells were starved in DMEM/F12 supplemented with 0.5% FCS for 24 h, then washed and resuspended in serum free DMEM/F12 at 4 × 10^7^ cells/ml. After 5 min pre-incubation at 37°C, the cells were stimulated with equal volume of vASC or ASC.B6 conditioned media for different time points. The stimulation was stopped by adding 2× lysis buffer [1 × lysis buffer contains: 50 mM TrisHCl pH 7.4, 150 mM NaCl, 2 mM EDTA, 20 mM NaF, 200 μM Na_3_VO_4_, 1 mM PMSF, cOmplete™ Mini EDTA-free Protease Inhibitor Cocktail (Roche)]. After 30 min lysis on ice the samples were centrifuged for 15 min at 13,000 × *g* and then the lysates were boiled with 2× sample loading buffer for 5 min. Cells lysates from 1.5 × 10^5^ cells were run on a 10% SDS-PAGE, and transferred to PVDF membranes. The membranes were blocked with 3% gelatin from cold-water fish skin in PBS for 1 h at room temperature, and then incubated with anti-phospho Akt (Ser473) antibody (Cell Signaling Technology, #9271) overnight at 4°C. After washing and incubating with swine anti-rabbit Ig-HRP (DAKO) for 1 h at room temperature, the immunoreactive proteins were visualized using WesternBright ECL HRP substrate (Advansta), and the chemiluminescence signal was detected with Odyssey Imaging System (LI-COR Biotechnology). To re-probe with different antibodies, the membranes were stripped in stripping buffer (Re-Blot Plus Strong, Millipore) for 15 min at room temperature. The amount of loaded proteins was tested with anti-Akt (Pan) antibody (Cell Signaling Technology, #2920), followed by anti-mouse Ig-HRP (DAKO).

### Proliferation Test

4T1 cells were starved in DMEM/F12 supplemented with 0.5% FCS for 24 h, then washed and seeded onto a 24 well plate at 5 × 10^4^ cells/well in DMEM/F12 supplemented with 2% FCS. ASC.B6 cells were washed in serum free DMEM/F12 and resuspended in DMEM/F12 + 2% FCS, then seeded in Transwell inserts with 0.4 μm pore size (Corning Costar), at a 4T1: ASC ratio of 2: 1, i.e., 2.5 × 10^4^ cells/insert. As a control, only DMEM/F12 + 2% FCS was pipetted into Transwell inserts. For blocking the proliferative effect of ASC.B6, recombinant mouse IGFBP2 (R&D Systems, 797-B2-025) was added at a concentration of 1 μg/ml in lower chambers. 4T1 cells were harvested after 48 h, and the living cell number of 4T1 was determined with trypan blue staining and counting with BioRad TC10 counter device.

### Statistical Analyses

Experiments were repeated at least three times each conducted in triplicate samples, unless indicated otherwise in the figure legends. Mean and SD were determined with Microsoft EXCEL software from the results of the three independent experiments. Statistical analysis were carried using *t*-test (set at ^*^P < 0.05, ^**^P < 0.01).

## Results

### ASC.B6 Increases Breast Cancer Progression and Metastasis

**Figure 1:**
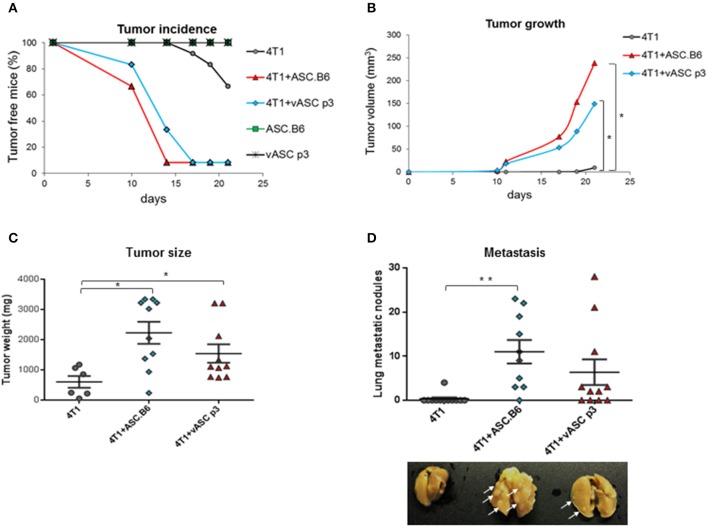
ASCs promote tumor initiation, growth and metastasis. 4T1 cells (10^3^ cells/mouse) were injected into BALB/c female mice with or without vASCs or ASC.B6 (10^5^ cells/mouse), control mice were injected with ASCs without tumor cells. *n* = 10–12. **(A)** Tumor initiation was regularly monitored and tumor incidence was evaluated using Kaplan-Meier analysis. **(B)** Tumor size was measured with a special caliper and its volume was determined as described in Material and Methods. **(C)** Mice were sacrificed on day 28 after tumor injection, and then primary tumors were resected and weighed. **(D)** The macroscopic metastatic nodules (white arrows) were counted on isolated lungs. The statistical analysis was *t*-test with *P*-values set at: *P < 0.05, **P < 0.01.

Adipose-tissue derived stem cells were reported to promote tumor growth (12). In our previous study, we have shown that expression of genes involved in cancer, cellular growth, proliferation and cellular movement changed significantly with polyploidization of ASCs (13). To compare the tumor-promoting activity of ASCs, we co-injected 4T1 murine breast cancer cells with vASCs at low passage number (p3) or with polyploid ASC.B6 cells into the mammary pad of female BALB/c mice. Neither vASCs nor ASC.B6 cells alone induced tumors (Figure 1A). 4T1 alone formed detectable tumors only after 2–3 weeks. However, palpable tumors appeared much earlier, within 10 days in ASC + 4T1 co-injected mice. ASC.B6 was more potent in facilitating tumor initiation, as more animals bore tumor in ASC.B6 co-injected mice at day 10 and 14 than in the vASC co-injected group. Both ASCs augmented the volume and weight of the tumors; however, tumors were bigger in ASC.B6 than vASC co-injected mice (Figures 1B,C). Finally, both ASCs increased the number of macroscopic metastatic nodules in the lung, but the number and size of these nodules was higher in case of ASC.B6 + 4T1 co-injected mice (Figure 1D). These results suggest that ASCs intensify breast cancer growth and metastasis, and this capability increases in ASC.B6 cells with abnormal karyotype.

### ASC.B6 Upregulates IGF1 and Downregulates IGFBP2 Expression to Promote Tumor-Cell Proliferation

**Figure 2:**
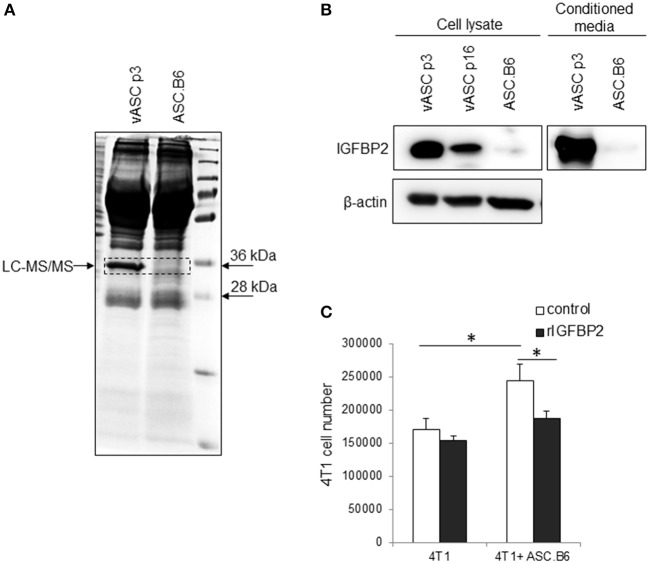
IGFBP2 expression of vASCs. **(A)** SDS-PAGE analysis is shown of the proteins precipitated from conditioned media of vASCs or ASC.B6 cell culture, stained with Coomassie Brilliant Blue R-250. **(B)** IGFBP2 protein was detected by Western blotting experiment from the cell lysate of vASCs at passage number p3 and p16, and ASC.B6 cell cultures or conditioned media of vASCs p3 and ASC.B6 cell cultures. β-actin was used as loading control. **(C)** Recombinant IGFBP2 (1 μg/ml) inhibited the proliferation of 4T1 cells in the presence of ASC.B6 at a ratio of 2.5:1 in Transwell inserts. The bars show the mean ± SD from three independent experiments, the statistical analysis was *t*-test with *P*-values set at: *P < 0.05.

Previously, we have shown that ASCs upregulated IGF1 expression through polyploidization and that the ASC-derived IGF1 promoted 4T1 proliferation (13). Searching for additional differentially expressed factors, we found an abundant protein in the supernatant of vASCs, which was absent from ASC.B6 (Figure 2A). Liquid chromatography-mass spectrometry analysis identified this 32 kDa protein as Insulin-like growth factor-binding protein 2 (IGFBP2), a member of the insulin-like growth factor regulating proteins (15). We confirmed by Western blotting that vASCs at p3 expressed and secreted huge amount of IGFBP2, vASCs at later passages (p16) decreased its expression, while ASC.B6 completely downregulated it (Figure 2B). 4T1 cells proliferated better in the presence of ASC.B6, however, adding recombinant IGFBP2 (rIGFBP2) to the cell culture, it resulted in significant decrease in ASC.B6 tumor-growth promoting effect (Figure 2C). Based on our results, we concluded that vASCs are normally regulating the accessibility of growth factors by secreting high amount of IGFBP2, however, the ratio of IGF1 and IGFBP2 changed in polyploid ASC.B6, leading to enhanced cell growth and survival.

### IGF1/IGFBP2 Balance the Survival Signaling Pathway of Breast Cancer Cells

**Figure 3:**
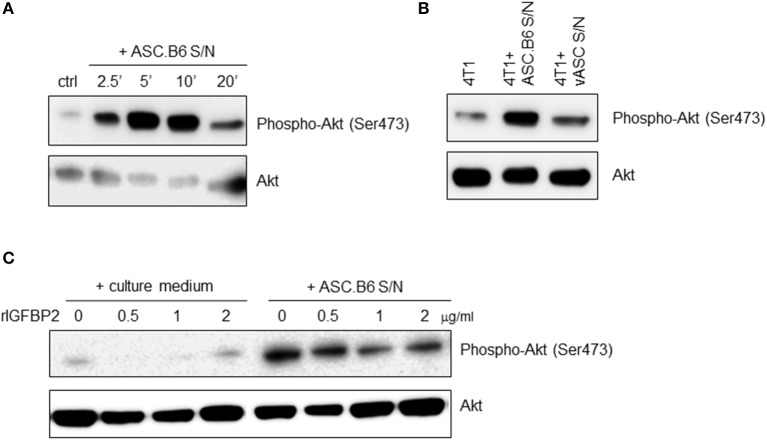
Secreted factors from ASC.B6 induce Akt phosphorylation in 4T1 cells. 4T1 cells were stimulated with conditioned media of vASCs and then the cells were lysed and the phosphorylated or total Akt proteins were detected in Western blotting experiments. **(A)** 4T1 cells were stimulated with conditioned medium of ASC.B6 at various time points (2.5, 5, 10, or 20 min) or only cell culture medium was added (ctrl). **(B)** 4T1 cells were stimulated with conditioned medium of ASC.B6 or vASCs for 5 min or left unstimulated. **(C)** Cell culture medium or conditioned medium of ASC.B6 were pre-incubated with rIGFBP2 in various concentrations (0.5, 1, 2 μg/ml) and then added to 4T1 cells for 5 min.

IGF1 is a general mitogen, it induces pro-survival signaling pathways, such as the Ras/MAPK and the PI3K/Akt kinase pathway (17). When we added ASC.B6 conditioned medium to 4T1 cells, it rapidly induced Akt phosphorylation, with a maximum at 5 min (Figure 3A). In contrast, supernatant of vASCs at p3 did not elevate substantially the level of phosphorylated Akt over the baseline, suggesting that soluble factors secreted by ASC.B6, such as IGF1, promoted the pro-survival cell response (Figure 3B). When we pre-incubated the ASC.B6 conditioned medium with rIGFBP2, it decreased the induction of Akt phosphorylation, while rIGFBP2 alone did not impact Akt phosphorylation (Figure 3C). Our results indicate that polyploid ASC.B6 cells activate the pro-survival pathway in 4T1 cancer cells by changing the balance of their paracrine factors, namely, by downregulating IGFBP2 and secreting IGF1.

## Discussion

In breast, mammary tissue is embedded in the adipose tissue, which allows direct paracrine action of adipose-derived factors on mammary epithelial cells; thereby the stromal cells contribute to the regulation of cellular growth and differentiation of the mammary gland (18). However, adipose tissue-derived cells may also be a source for tumor stroma (12), and their secreted growth factors, especially IGF1, contribute to the growth of breast cancer cells (19). In this way, ASCs may play crucial role in the development of breast cancer and in the relapse of the disease as well. Importantly, adipose tissue and enriched ASCs are often used for reconstruction of breast tissue after mastectomy (20), which carries the risk that residual cancer cells might be activated by the stem cells, leading to fatal outcome. Our results point out, that ASCs can robustly augment tumor formation even when low number of tumor cells is present, and they facilitate the progression of the disease, leading to rapid metastatic events. When adipose stem cells go wrong, they change their transcriptome in a way, which may favor cancer development. We have previously shown that transcription of more than 2,000 genes changed in polypoid ASC.B6 cells compared to vASCs with normal karyotype, amongst them there are genes, which are involved in the regulation of cancer, cellular growth and proliferation (13). In addition, we found that ASC.B6 cells promoted 4T1 cell proliferation better than normal vASCs, suggesting that they gained stronger tumor-promoting function. In our present *in vivo* experiment, we verified that polyploid ASC.B6 cells facilitated tumor progression better than vASCs with normal karyotype. The explanation for the increase in efficacy may be the massive production of IGF1 by ASC.B6 cells, which contributes to the 4T1 cell proliferation in an *in vitro* assay (13). We show here that ASC.B6-derived factors induce Akt phosphorylation, hence switch on the PI3K/Akt signaling pathway, and thereby promote cell survival, proliferation, growth and cellular metabolic pathways (17, 21) In addition, we have identified IGFBP2 in the secretome of vASCs, which sequesters IGF1 and thereby regulates its accessibility and function (15). IGFBP2 was missing from ASC.B6 cells, and supplementation with a recombinant protein abolished tumor cell proliferation, and also mitigated the ASC.B6-induced Akt phosphorylation. Our results indicate that the tumor-growth promoting function of ASCs is influenced by the balance of IGF1/IGFBP2.

It is well-known that IGF1 is a key growth factor in mammary gland formation during development, however, it also plays important role in breast cancer (14). There are anti-cancer therapies under clinical trials which target the IGF/IGF-receptor system, however, most of them failed due to interfering with insulin signaling and manifesting metabolic toxicity (22). In contrast, IGFBPs can prevent IGF from binding to IGF-1R, but do not bind insulin and thus do not interfere with insulin-insulin receptor interactions; therefore they might be promising therapeutic targets. However, there are conflicting results whether IGFBP2 is tumor suppressive or oncogenic. Interestingly, IGFBP2 is often upregulated in various cancer types, such as gliomas, prostate, ovarian, and breast cancer (16). The oncogenic activity of IGFBP2 can be explained by its IGF-independent activities: it possesses a functional integrin-binding domain, heparin binding domains and a nuclear localization signal motif as well, all contributing to cellular signaling leading to cell proliferation, migration, and angiogenesis (23). In case of benign proliferative breast diseases and various type of breast cancers, the level of IGFBP2 both in serum and tumor mass may be upregulated (24, 25), moreover, IGFBP2 has proliferative effect on breast cancer cell lines (26) and it is associated with the endocrine resistance of breast cancer (27), which suggests that IGFBP2 is oncogenic and it is a potential biomarker (16). Based on IGFBP2 positivity of breast cancers, a DNA vaccine was designed, encoding HER-2/neu, IGFBP2 and insulin-like growth factor 1 receptor (IGF1R) as tumor associated antigens and it proved to be effective in preclinical trials. It blocks the development of palpable lesions and slows tumor growth in TgMMTV-neu mice, which develop spontaneous mammary cancer expressing these antigens (28). Currently, a phase I clinical trial is going on with pUMVC3 vector and IGFBP2, HER2, and IGF1R to study the side effects and to determine the best dose of a vaccine therapy in preventing cancer recurrence in patients with non-metastatic, node positive, HER2 negative breast cancer (Clinicaltrials.gov ID: NCT02780401).

In contrast to these studies, IGFBP2 behaves as tumor suppressor in our experiments: it inhibits the IGF1-induced proliferation and the pro-survival PI3K/Akt pathway in 4T1 cells. There are similar results in the literature: IGFBP2 inhibits the proliferation of human breast cancer cell line Hs578T (29), which does not express IGFBP2, similarly to 4T1 (data not shown), suggesting that the pro- or anti-tumorigenic effect of IGFBP2 depends on the actual state of the cancer cell. It was also assumed that the immunohistochemical methods used for the detection of IGFBP2 in tumor sections recognized its cleaved forms as well. The cleaved IGFBP2 has reduced affinity for IGFs, allowing their functions through IGF1R. Indeed, the protease-resistant IGFBP2 inhibits MCF-7 human breast cancer cell proliferation *in vitro*, and when it is combined with a non-matrix-binding mutation, the IGFBP2 mutant more effectively inhibits the growth and angiogenesis of MCF-7 tumor xenograft *in vivo* (30). In addition, expression of IGFBP2 is influenced by various conditions, such as the body mass index (BMI), diet, physical activity, age and hormonal status. A recent study from a large series of primary invasive breast cancers showed that tumor expression of IGFBP2 was a positive predictor of overall survival in a multivariate analyses adjusted for BMI, and its expression correlated with estrogen receptor status (31). Similarly, serum IGFBP2 is associated with a decrease in risk of atypical hyperplasia in the age- and BMI adjusted model and non-users of hormone therapy (32). The contradiction in literature data warns on that the IGF1/IGFBP2 system is delicately regulated and the outcome of the signaling depends on numerous factors, which have to be taken in account during therapy design.

## Conclusions

Accumulating data suggest that ASCs play important role in the organization of breast tissue, and when their secreted factors change, it may lead to pathological processes. Our results show that ASCs with altered genetic background promote tumor progression by unbalanced IGF1 and IGFBP2 secretion, which leads to enhanced growth of breast epithelial cells and favors tumorigenesis. We suggest that our model may be suitable to further search for new players responsible for breast cancer development, and to find new therapeutic targets in tumor stroma.

## Data Availability Statement

The datasets generated for this study are available on request to the corresponding author.

## Ethics Statement

The animal experiments were performed in accordance with animal experimentation and ethics guidelines of the EU (2010/63/EU). Experimental protocols were approved by the Review Committee of Biological Research Centre and by the Joint Local Ethics and Animal Welfare Committee of Avidin Ltd., then by National Food Chain Safety Office Directorate of Animal Health and Animal Welfare (NÉBIH) in possession of an ethical clearance XXIX./128/2013.

## Author Contributions

RF-B participated in the design of the study, performed experiments, analyzed the data, prepared the figures, and helped to draft the manuscript. GS and ÉH-G performed experiments and analyzed the data. LP revised the manuscript. RK designed the research, analyzed the data, and wrote the paper. All contributors meet the criteria for authorship.

### Conflict of Interest

LP was employed by the company Avidin Ltd. The remaining authors declare that the research was conducted in the absence of any commercial or financial relationships that could be construed as a potential conflict of interest.

## Abbreviations

Akt/PKB: protein kinase B
ASCs: adipose-tissue stem cells
IGF1: insulin-like growth factor 1
IGFBP2: insulin like growth factor–binding protein 2
MSC: mesenchymal stem cells
vASC: visceral adipose-tissue stem cells.
